# Dynamical Quantum Filtering via Enhanced Scattering of *para*-H_2_ on the Orientationally Anisotropic Potential of SrTiO_3_(001)

**DOI:** 10.1038/s41598-020-62605-8

**Published:** 2020-04-03

**Authors:** Koji Shimizu, Wilson Agerico Diño, Hiroshi Nakanishi, Hideaki Kasai, Kotaro Takeyasu, Katsuyuki Fukutani, Ayako Yajima

**Affiliations:** 1 Department of Applied Physics, Osaka University, Osaka, 565-0871 Japan; 2 Center for Atomic and Molecular Technologies, Osaka University, Osaka, 565-0871 Japan; 3 National Institute of Technology, Akashi College, Hyogo, 674-8501 Japan; 4 Institute of Industrial Science, The University of Tokyo, Tokyo, 153-8505 Japan; 5 Kawasaki Heavy Industries, Ltd., Hyogo, 673-8666 Japan

**Keywords:** Two-dimensional materials, Surfaces, interfaces and thin films

## Abstract

Quantum dynamics calculation, performed on top of density functional theory (DFT)-based total energy calculations, show dynamical quantum filtering via enhanced scattering of *para*-H_2_ on SrTiO_3_(001). We attribute this to the strongly orientation-dependent (electrostatic) interaction potential between the H_2_ (induced) quadrupole moment and the surface electric field gradient of ionic SrTiO_3_(001). These results suggest that ionic surfaces could function as a scattering/filtering media to realize rotationally state-resolved H_2_. This could find significant applications not only in H_2_ storage and transport, but also in realizing materials with pre-determined characteristic properties.

## Introduction

**Figure 1:**
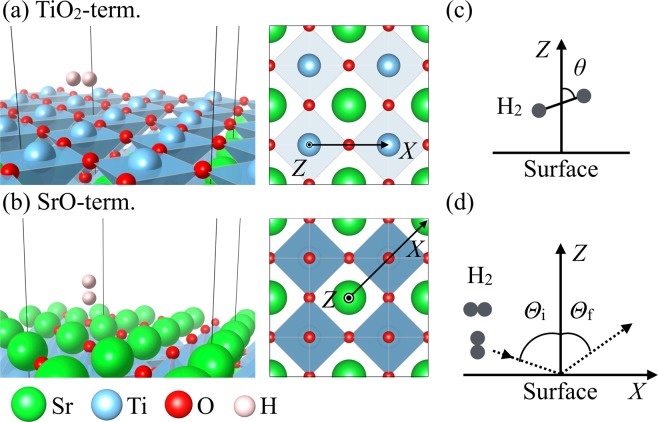
A depiction of H_2_ adsorption (**a**) atop the Ti-site and (**b**) atop the O-site on the TiO_2_- and SrO-terminated SrTiO_3_(001), with corresponding preferential orientations *θ* = *π*/2 and 0, respectively. The lower left panel shows which colored balls correspond to which element. (**c**) A depiction of H_2_ with the H_2_ center-of-mass (CM) at a distance *Z* from the surface, and the H-H bond at an orientation *θ* with respect to the surface normal. (**d**) A depiction of H_2_ scattering on SrTiO_3_(001) with angle of incidence *Θ*_i_ and scattering angle *Θ*_f_. *X* corresponds to the surface lateral position of the H_2_ CM along the (**a**) [100] and (**b**) [110] direction on the TiO_2_- and SrO-terminated SrTiO_3_(001), respectively.

The behavior/dynamics of H_2_ on surfaces strongly depend on the H_2_ molecular orientation/rotational states (rotational quantum number *J*, magnetic quantum number *m*) (cf., e.g., ref. ^1^ and references therein). On transition metal surfaces such as copper (Cu) and palladium (Pd), H_2_ exhibits rotational alignment (cf., e.g., refs. ^1,2^ and references therein). One could then tune or design the structure of the metal (alloy) surfaces to dynamically filter the quantum rotational states of desorbing or scattered H_2_ (dynamical quantum filtering), and control the H_2_ dynamics (cf., e.g., refs. ^1–7^ and references therein). The resulting H_2_ dynamics could, in turn, be used to probe local surface reactivity^8,9^ (e.g., via the H_2_(D_2_) diffraction spectra^10–12^). On ionic crystal surfaces, the H_2_ quadrupole moment interacts with the surface local electric field gradient to couple the translational and rotational degrees-of-freedom^13^. On SrTiO_3_(001) (STO(001)), an ionic crystal material^14–21^ with tunable surface terminations (cf., e.g., refs. ^22,23^), H_2_ adsorbs with the H-H bond oriented parallel (polar coordinate *θ* = *π*/2) to the TiO_2_-terminated surface at the Ti-site, and perpendicular (*θ* = 0) to the SrO-terminated surface on top of the O-site (cf., Fig. 1)^24^. The strong orientationally anisotropic potential ($\Delta {E}_{{\rm{anisotropy}}}^{\theta }=| {E}_{\theta =0}-{E}_{\theta =\pi /2}|$) results in adsorbed H_2_ with (hindered) rotational states (*J*, *m*) different from that of gas phase H_2_. These strongly hindered adsorption states lead to (*J*, *m*)-dependent thermal desorption energies^24–30^, suggesting the possibility of separating para-H_2_ [*p*-H_2_(*J* = 0, *m* = 0)] and ortho-H_2_ [*o*-H_2_(*J* = 1, *m* = ±1)] through an adsorption-desorption process. This could find significant applications not only in H_2_ storage and transport applications, but also in realizing materials with pre-determined characteristic properties.

As with metal (alloy) surfaces^5,8,9^, the H_2_ dynamics would be susceptible to the positive and negative charges that corrugate ionic crystal surfaces. In the following, we will show that on STO(001), under the influence of the orientationally anisotropic potential, on top of the surface lateral corrugation, *p*-H_2_ scatter strongly at specific angles from the TiO_2_-terminated and SrO-terminated STO(001). This dynamical filtering/scattering selectivity allows for more economical (less heat consumption) and more efficient means to rotationally separate *o*-H_2_ and *p*-H_2_, than the usual adsorption-desorption process^1–5,24–30^.

## Results and Discussion

### H_2_/SrTiO_3_(001) System

Figure 1 shows a H_2_ interacting with STO(001). *X* gives the surface lateral coordinate of the H_2_ center-of-mass (CM) along the most corrugated directions on the two STO(001) terminations, viz., along [100] for TiO_2_-termination (Ti-O-Ti row) and along [110] for SrO-termination (Sr-O-Sr row), respectively (cf., Fig. 1(a,b)). *Z* gives the normal distance of the H_2_ CM from the surface. *θ* gives the polar angular orientation of the H-H bond with respect to the surface normal. *ϕ* (not shown) gives the azimuthal angular orientation of the H-H bond about the surface normal, with respect to the *X*-axis, at each site on STO(001). *Θ*_i(f)_ gives H_2_ incidence (scattering) angle measured with respect to the surface normal (cf., Fig. 1(c,d)). In the following, given H_2_(*J*_i_, *m*_i_, *E*_i_, *Θ*_i_), we determine the probability of finding H_2_(*J*_f_, *m*_f_, *E*_f_, *Θ*_f_). H_2_(*J*_i_, *m*_i_, *E*_i_, *Θ*_i_) indicates a H_2_ with initial rotational state (*J*_i_, *m*_i_), impinging STO(001) with an initial incident translational energy *E*_i_ and at an incidence angle *Θ*_i_ with respect to the surface normal. H_2_(*J*_f_, *m*_f_, *E*_f_, *Θ*_f_) indicates a H_2_ with final rotational state (*J*_f_, *m*_f_), scattered from STO(001) with a final translational energy *E*_f_ and at a scattering angle *Θ*_f_ with respect to the surface normal.

### Orientationally anisotropic electrostatic potential

**Figure 2:**
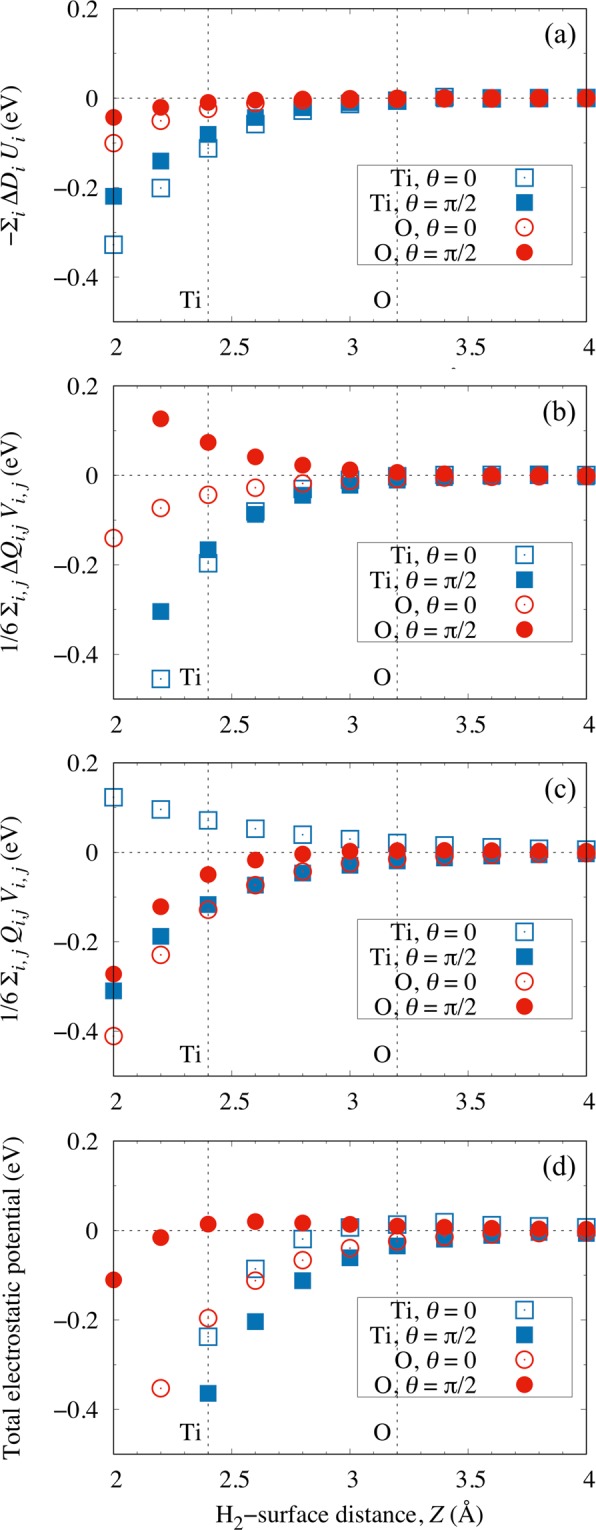
Calculated electrostatic potential contributions from (**a**) the induced dipole moment Δ*D*_*i*_ with the surface electric field *U*_*i*_, (**b**) the induced quadrupole moment Δ*Q*_*i*,*j*_ and (**c**) the H_2_ quadrupole moment *Q*_*i*,*j*_ with the gradient of the surface electric field *V*_*i*,*j*_, on the Ti-site (squares) and the O-site (circles) with *θ* = 0 (open symbols) and *θ* = *π*/2 (filled symbols) for the TiO_2_-terminated STO(001). (**d**) The sum of the contributions (**a**–**c**). *i*, *j* = (*x*, *y*, *z*). The dotted vertical lines intercepting the abscissas indicate locations of the corresponding potential energy minima (H_2_-surface equilibrium distance) at each respective surface site.

**Figure 3:**
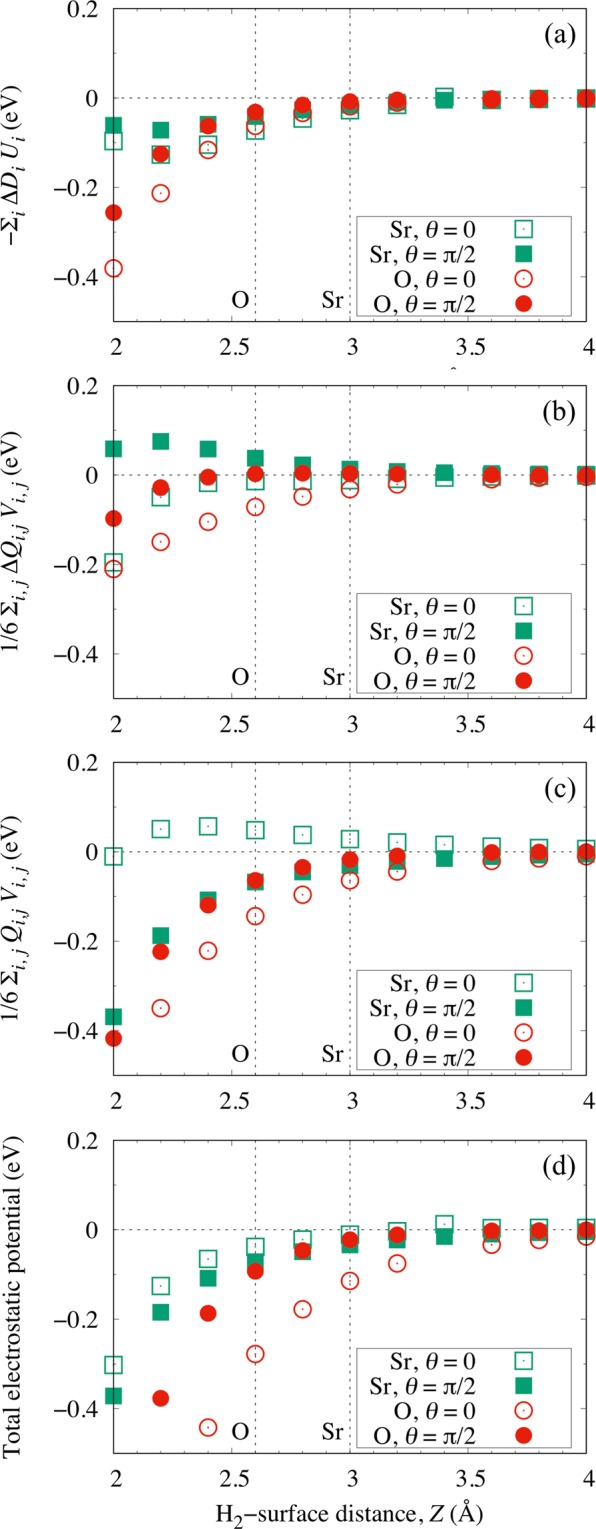
Calculated electrostatic potential contributions from (**a**) the induced dipole moment Δ*D*_*i*_ with the surface electric field *U*_*i*_, (**b**) the induced quadrupole moment Δ*Q*_*i*,*j*_ and (**c**) the H_2_ quadrupole moment *Q*_*i*,*j*_ with the gradient of the surface electric field *V*_*i*,*j*_, on the Sr-site (squares) and the O-site (circles) with *θ* = 0 (open symbols) and *θ* = *π*/2 (filled symbols) for the SrO-terminated STO(001). (**d**) The sum of the contributions (**a**–**c**). *i*, *j* = (*x*, *y*, *z*). The dotted vertical lines intercepting the abscissas indicate the locations of the corresponding potential energy minima (H_2_-surface equilibrium distance) at each respective surface site.

In Figs. 2 and 3, we plot the (electrostatic interaction energies) dot products of the (induced) dipole moment with the surface electric field *U*_*i*_ and the (induced) quadrupole moment with the gradient of the surface electric field *V*_*i*,*j*_, for the TiO_2_-terminated and SrO-terminated surfaces, respectively. We can see that the orientational (*θ*) anisotropy $\Delta {E}_{{\rm{anisotropy}}}^{\theta }$ becomes important when the impinging H_2_ comes sufficiently near the surface, viz., at *Z* ≤ 2.4 Å above the Ti-site and *Z* ≤ 2.6 Å above the O-site on the TiO_2_- and SrO-terminated surfaces, respectively. On the other hand, far from the surface, viz., at *Z* ≥ 3.2 Å above the O-site and *Z* ≥ 3.0 Å above the Sr-site of the TiO_2_- and SrO-terminated surfaces, respectively, only a small $\Delta {E}_{{\rm{anisotropy}}}^{\theta }$ can be observed. This orientational anisotropy $\Delta {E}_{{\rm{anisotropy}}}^{\theta }$, on top of the surface lateral corrugation, would prove to be useful in our attempt to control the H_2_ scattering dynamics, as we will discuss in detail in the next sections.

### H_2_ Scattering along STO(001)[100] on TiO_2_-terminated STO(001)

**Figure 4:**
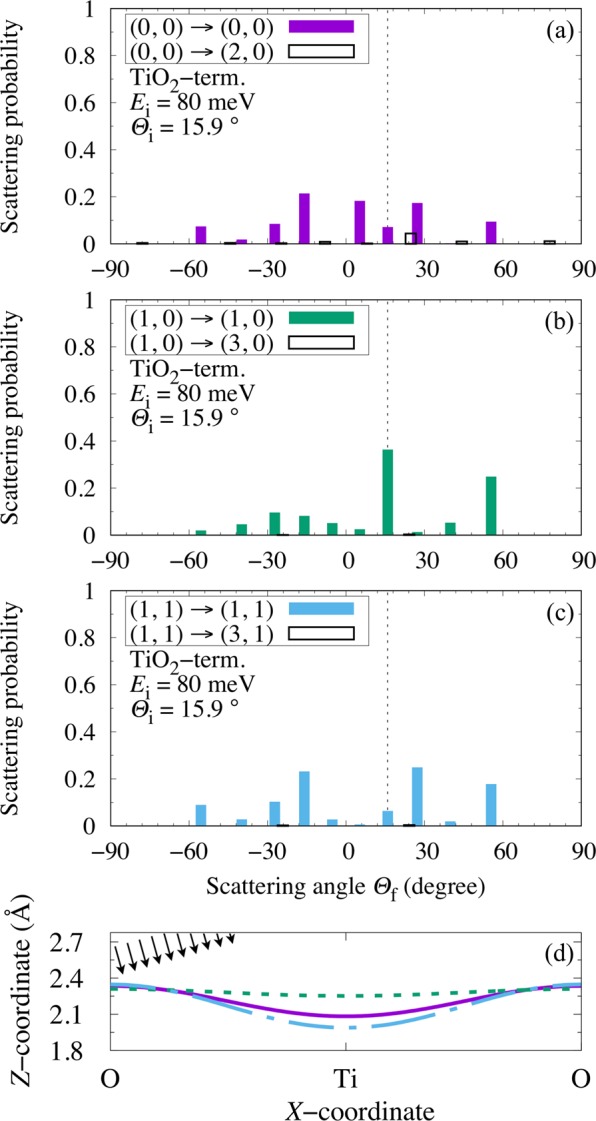
(**a**–**c**) Calculated H_2_ scattering probability (*J*_i_, ∣*m*_i_∣) → (*J*_f_, ∣*m*_f_∣) from initial rotational state (*J*_i_, ∣*m*_i_∣) to final rotational state (*J*_f_, ∣*m*_f_∣) on the TiO_2_-terminated STO(001) as a function of the scattering angle *Θ*_f_, for incident energy *E*_i_ = 80 meV and incident angle *Θ*_i_ = 15.9°. The dotted vertical lines intercepting the abscissas indicate specular scattering. (**d**) Solid, dotted, and dash-dot lines indicate constant energy surfaces of $\langle {Y}_{J}^{m}| V(Z,\theta ,X)| {Y}_{J}^{m}\rangle$ (with 80 [meV] $\times {\cos }^{2}{\varTheta }_{{\rm{i}}}$: corresponding to H_2_ surface normal translational energy) encountered by a H_2_ impinging with initial rotational states (*J*_i_, *m*_i_): [(0, 0), (1, 0), (1, 1)], respectively. Same scales used for *X*- and *Z*-coordinates. The arrows indicate the direction of incidence of the impinging H_2_.

In Fig. 4(a–c), we show the calculated (scattering) probabilities/spectra ${P}_{{J}_{{\rm{i}}}\to {J}_{{\rm{f}}}}^{{m}_{{\rm{i}}}\to {m}_{{\rm{f}}}}({E}_{{\rm{i}}}=80\,{\rm{meV}},{E}_{{\rm{f}}},{\varTheta }_{{\rm{i}}}={15.9}^{\circ },{\varTheta }_{{\rm{f}}})$ of finding H_2_(*J*_i_, *m*_i_, *E*_i_ = 80 meV, *Θ*_i_ = 15.9°) scattered as H_2_(*J*_f_, *m*_f_, *E*_f_, *Θ*_f_) along the [100] direction of a TiO_2_-terminated STO(001). (The corresponding initial surface perpendicular translational energy ${E}_{{\rm{i}}}\times {\cos }^{2}{\varTheta }_{{\rm{i}}}=80\,{\rm{meV}}\times {\cos }^{2}({15.9}^{\circ }) \sim 74.0$ meV and initial surface lateral translational energy ${E}_{{\rm{i}}}\times {\sin }^{2}{\varTheta }_{{\rm{i}}}=80\,{\rm{meV}}\times {\sin }^{2}({15.9}^{\circ }) \sim 6.00$ meV). This corresponds to the maximum *p*-H_2_ to *o*-H_2_ ratio *R*_*J*=0/*J*=1_(*E*_i_ = 80 meV, *Θ*_i_ = 15.9°, *Θ*_f_) on a TiO_2_-terminated STO(001) (cf., Table 1). The resulting trends can be explained by inspecting the corresponding orientational anisotropy and surface lateral corrugation of the potential encountered by the impinging H_2_(*J*_i_, *m*_i_, *E*_i_ = 80 meV, *Θ*_i_ = 15.9°) (i.e., $\langle {Y}_{J}^{m}| V(Z,\theta ,X)| {Y}_{J}^{m}\rangle$, cf., Fig. 4(d)). Note that along STO(001)[100], H_2_(*J*_i_ = 0, *m*_i_ = 0) encounters a strongly corrugated and orientationally anisotropic PES (cf., solid curve in Fig. 4(d)), that favors molecular adsorption atop the Ti-site, with H-H bond oriented parallel to the surface, and an adsorption energy *E*_ads_ = − 191 meV^24^. Atop the O-site, H_2_ preferentially adsorbs with the H-H bond oriented perpendicular to the surface, with *E*_ads_ = − 72.5 meV^24^. As a result, in Fig. 4(a), we find a corresponding spectra dominated by off-specularly scattered H_2_, with dominant components coming from backscattering. Most of the H_2_(*J*_i_ = 0, *m*_i_ = 0) would be molecularly adsorbed (due to *reorientation/steering*^31,32^), and only a small fraction would be elastically scattered (due to *shadow effect*^9^). Considering the angle of incidence, the inelastically scattered H_2_ would come from those hitting the repulsive part of the potential well near the O-site (*shadow effect*, cf., solid curve in Fig. 4(d)), resulting in a change in surface lateral momentum and off-specular scattering angles. Energy transfer from the translational degree-of-freedom (DOF) to the rotational DOF allows the molecule more time to sample the anisotropic surface through *reorientation/steering*. Those that succeed would molecularly adsorb. Those that fail, would be rotationally de-excited on the way back to the gas phase, because of the smaller anisotropic potential further (out in the vacuum) from the surface. Thus, the scattering spectra shows negligible rotationally excited H_2_, i.e., H_2_(*J*_f_ = 2, *m*_i_ = 0). H_2_(*J*_i_ = 1, *m*_i_ = ±1) also encounters a strongly anisotropic PES (cf., dash-dot curve in Fig. 4(d)). Thus, we see the same trend in Fig. 4(c), i.e., a corresponding spectra dominated by off-specularly scattered H_2_, with dominant components coming from backscattering. On the other hand, H_2_(*J*_i_ = 1, *m*_i_ = 0) encounters an almost flat PES (cf., dotted curve in Fig. 4(b)). As a result, we see strong (dominant) specular scattering of H_2_(*J*_i_ = 1, *m*_i_ = 0) (Fig. 4(b)). Finally, we could observe a *p*-H_2_ to *o*-H_2_ ratio as large as ca. 4.96 (cf., Table 1) compared to that of *normal*-H_2_ (*n*-H_2_) (i.e., >1/3 at 300 K) at scattering angle *Θ*_f_ = 5.26° (Fig. 4).

**Table 1:** Calculated *R*_*J*=0/*J*=1_(*E*_i_, *Θ*_i_, *Θ*_f_) and $\bar{{R}_{J=0/J=1}}({E}_{{\rm{i}}},{\varTheta }_{{\rm{i}}},{\varTheta }_{{\rm{f}}})$ on TiO_2_-terminated STO(001) at the incident energy range 10 meV ≤ *E*_i_ ≤ 80 meV. For each incident energy, we list only the maximum *R*_*J*=0/*J*=1_(*E*_i_, *Θ*_i_, *Θ*_f_) with the corresponding incident and scattering angles.

*E*_i_ [meV]	*Θ*_i_[°]	*Θ*_f_[°]	*R*_*J*=0/*J*=1_	$\bar{{R}_{{\boldsymbol{J}}{\boldsymbol{=}}{\bf{0}}/{\boldsymbol{J}}{\boldsymbol{=}}{\bf{1}}}}$
10.0	15.0	15.0	1.45	0.470
20.0	47.1	0.00	1.91	0.155
30.0	48.4	48.4	1.14	0.397
40.0	40.4	−7.44	1.92	0.0967
50.0	20.3	6.65	2.65	0.449
60.0	18.5	31.9	1.70	0.0753
70.0	43.3	43.3	1.64	0.265
80.0	15.9	5.26	4.96	0.271

### H_2_ Scattering along STO(001)[110] on SrO-terminated STO(001)

**Figure 5:**
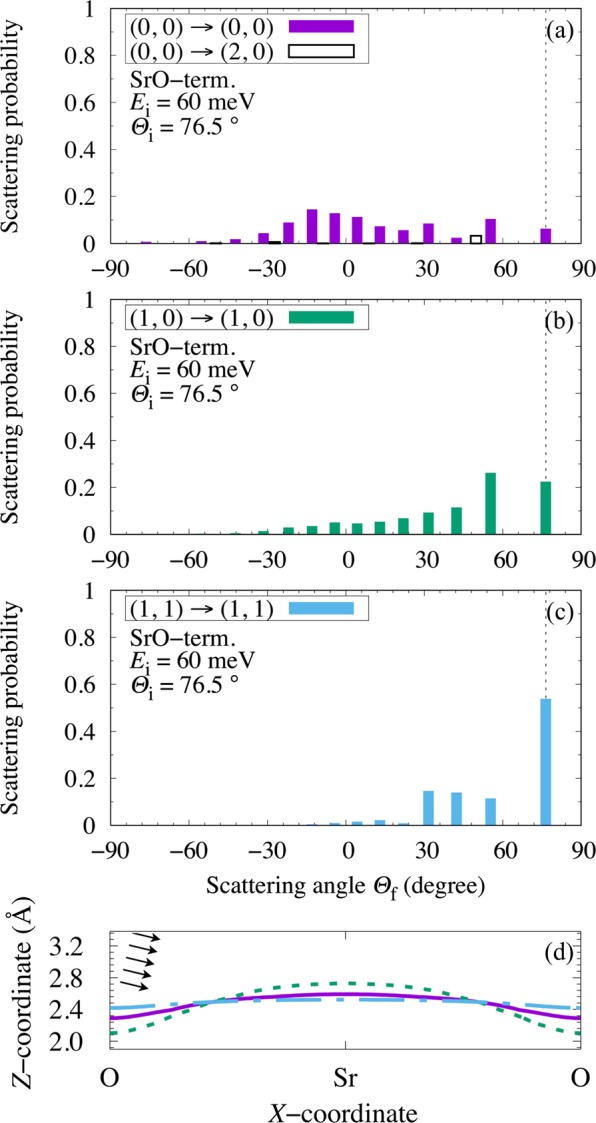
(**a**–**c**) Calculated H_2_ scattering probability (*J*_i_, ∣*m*_i_∣) → (*J*_f_, ∣*m*_f_∣) from initial rotational state (*J*_i_, ∣*m*_i_∣) to final rotational state (*J*_f_, ∣*m*_f_∣) on the SrO-terminated STO(001) as a function of the scattering angle *Θ*_f_, for incident energy *E*_i_ = 60 meV and incident angle *Θ*_i_ = 76.5°. The dotted vertical lines intercepting the abscissas indicate specular scattering. (**d**) Solid, dotted, and dash-dot lines indicate constant energy surfaces of $\langle {Y}_{J}^{m}| V(Z,\theta ,X)| {Y}_{J}^{m}\rangle$ (with 60 [meV] $\times {\cos }^{2}{\varTheta }_{{\rm{i}}}$: corresponding to H_2_ surface normal translational energy) encountered by a H_2_ impinging with initial rotational states (*J*_i_, *m*_i_): [(0, 0), (1, 0), (1, 1)], respectively. Same scales used for *X*- and *Z*-coordinates. The arrows indicate the direction of incidence of the impinging H_2_.

In Fig. 5(a–c), we show the calculated (scattering) probabilities/spectra corresponding to the maximum *para*-to-*ortho* ratio *R*_*J*=0/*J*=1_(*E*_i_ = 60 meV, *Θ*_i_ = 76.5°, *Θ*_f_), along the [110] direction of a SrO-terminated STO(001) (cf., Table 2). (The initial surface perpendicular translational energy ${E}_{{\rm{i}}}\times {\cos }^{2}{\varTheta }_{{\rm{i}}}=60\,{\rm{meV}}\times {\cos }^{2}({76.5}^{\circ }) \sim 3.27\,{\rm{meV}}$ and the initial surface lateral translational energy ${E}_{{\rm{i}}}\times {\sin }^{2}{\varTheta }_{{\rm{i}}}=60\,{\rm{meV}}\times {\sin }^{2}({76.5}^{\circ }) \sim 56.7\,{\rm{meV}}$.) Again, the resulting trends can be explained by inspecting the corresponding orientational anisotropy and surface lateral corrugation of the potential encountered by the impinging H_2_(*J*_i_, *m*_i_, *E*_i_ = 60 meV, *Θ*_i_ = 76.5°) (i.e., $\langle {Y}_{J}^{m}| V(Z,\theta ,X)| {Y}_{J}^{m}\rangle$, cf., Fig. 5(d)). Note that along STO(001)[110] on the SrO-terminated STO(001), H_2_(*J*_i_ = 0, *m*_i_ = 0) encounters a strongly corrugated and orientationally anisotropic PES (cf., solid curve in Fig. 5(d)), that now favors molecular adsorption atop the O-site, with H-H bond oriented perpendicular to the surface, and an adsorption energy *E*_ads_ = −151 meV^24^. Atop the Sr-site, H_2_ adsorbs with the H-H bond oriented parallel to the surface, and *E*_ads_ = −111 meV^24^. As a result, in Fig. 5(a), we find a corresponding spectra dominated by off-specularly scattered H_2_, with dominant components coming from backscattering. Again, most of the H_2_(*J*_i_ = 0, *m*_i_ = 0) would be molecularly adsorbed (due to *reorientation/steering*^31,32^), and only a small fraction would be elastically scattered (possibly due to *shadow effect*^9^). Considering the angle of incidence, the inelastically scattered H_2_ would come from those hitting the repulsive part of the potential well near the Sr-site (*shadowing effect*, cf., solid curve in Fig. 4(d)), resulting in a change in surface lateral momentum and off-specular scattering angles. Note that the impinging H_2_ has a larger surface lateral momentum as compared to the corresponding surface perpendicular component. This allows the impinging H_2_ more time to explore the anisotropic surface. Those that succeed would molecularly adsorb via *reorientation/steering*. Those that fail, would be rotationally de-excited on the way back to the gas phase, because of the smaller anisotropic potential further (out in the vacuum) from the surface. Thus, the scattering spectra shows negligible rotationally excited H_2_, i.e., H_2_(*J*_f_ = 2, *m*_i_ = 0). H_2_(*J*_i_ = 1, *m*_i_ = ± 1) also encounters a strongly anisotropic PES (cf., dash-dot curve in Fig. 5(d)). But now recall that the preferred adsorption site is at the O-site, with the H-H bond oriented perpendicular to the surface. Thus, we see strong specular scattering in Fig. 5(c). H_2_(*J*_i_ = 1, *m*_i_ = 0) shows higher backscattering probabilities (cf., Fig. 5(b)) due to the larger surface anisotropy along *X*, making it more susceptible to reorientation/steering. Finally, we could observe a *p*-H_2_ to *o*-H_2_ ratio as large as ca. 16.1 (cf., Table 2) compared to that of *n*-H_2_ (i.e., >1/3 at 300 K) at scattering angle *Θ*_f_ = − 76.5° (Fig. 5).

**Table 2:** Calculated *R*_*J*=0/*J*=1_(*E*_i_, *Θ*_i_, *Θ*_f_) and $\bar{{R}_{J=0/J=1}}({E}_{{\rm{i}}},{\varTheta }_{{\rm{i}}},{\varTheta }_{{\rm{f}}})$ on SrO-terminated STO(001) at the incident energy range 10 meV ≤ *E*_i_ ≤ 80 meV. For each incident energy, we list only the maximum *R*_*J*=0/*J*=1_(*E*_i_, *Θ*_i_, *Θ*_f_) with the corresponding incident and scattering angles.

*E*_i_[meV]	*Θ*_i_[°]	*Θ*_f_[°]	*R*_*J*=0/*J*=1_	$\bar{{R}_{{\boldsymbol{J}}{\boldsymbol{=}}{\bf{0}}/{\boldsymbol{J}}{\boldsymbol{=}}{\bf{1}}}}$
10.0	0.00	47.1	1.32	0.124
20.0	7.44	22.9	5.97	0.496
30.0	47.8	−47.8	11.4	0.529
40.0	15.9	−55.5	4.06	0.137
50.0	47.5	47.5	2.90	0.541
60.0	76.5	−76.5	16.1	0.0294
70.0	7.96	56.2	1.57	0.0194
80.0	57.3	−57.3	1.73	0.00328

### (Normalized) *p*-H_2_ to *o*-H_2_ Ratios

In Tables 1 and 2, we show the calculated *p*-H_2_ to *o*-H_2_ ratio *R*_*J*=0/*J*=1_(*E*_i_, *Θ*_i_, *Θ*_f_) and the normalized *p*-H_2_ to *o*-H_2_ ratio $\bar{{R}_{J=0/J=1}}({E}_{{\rm{i}}},{\varTheta }_{{\rm{i}}},{\varTheta }_{{\rm{f}}})$ of H_2_ scattered from the TiO_2_-terminated and SrO-terminated STO(001), respectively. The incident energy ranges from 10 meV ≤ *E*_i_ ≤ 80 meV. Assuming Maxwell-Boltzmann distribution, at 300 K, 90% of the impinging H_2_ would have kinetic energies less than 80 meV. In general, the SrO-terminated STO(001) show a much higher *p*-H_2_ yield compared to the TiO_2_-terminated STO(001). On TiO_2_-terminated STO(001), we observe a maximum *p*-H_2_ to *o*-H_2_ ratio of ${R}_{J=0/J=1}^{\max }=4.96$ for *E*_i_ = 80.0 meV, *Θ*_i_ = 15.9°, *Θ*_f_ = 5.26°. Whereas on SrO-terminated STO(001), we observe a maximum *p*-H_2_ to *o*-H_2_ ratio of ${R}_{J=0/J=1}^{\max }=16.1$ for *E*_i_ = 60 meV, *Θ*_i_ = 76.5°, *Θ*_f_ = − 76.5°. The results suggest that, for all incident energies *E*_i_ considered, the *p*-H_2_ to *o*-H_2_ ratios at specific scattering angles *Θ*_f_ exceed that of *n*-H_2_, i.e., *R*_*J*=0/*J*=1_(*E*_i_, *Θ*_i_, *Θ*_f_) > 1/3, at room temperature (*T* = 300 K).

## Conclusion

Here, we reported increased *para*-H_2_ (*p*-H_2_) to *ortho*-H_2_ (*o*-H_2_) ratio of H_2_ scattered from SrTiO_3_(001) (viz., ca. 4.96 and 16.1 along the SrTiO_3_(001)[100] and SrTiO_3_(001)[110] of the TiO_2_- and SrO-terminated SrTiO_3_(001), respectively). For reference, *normal*-H_2_ (*n*-H_2_) have a *p*-H_2_ to *o*-H_2_ ratio of 1/3, at room temperature (*T* = 300 K). We attribute this to the strongly orientation-dependent (electrostatic) interaction potential between the H_2_ (induced) quadrupole moment and the surface electric field of ionic SrTiO_3_(001). These results suggest that ionic surfaces (with tunable surface terminations) could function as a scattering/filtering media to realize rotationally state-resolved H_2_. This could find significant applications not only in H_2_ storage and transport, but also in realizing materials with pre-determined characteristic properties.

We can compare the present results to previous reports for the inelastic scattering of H_2_ on LiF(001), at normal incidence (*Θ*_i_ = 0°) and incident energy of *E*_i_ = 100 meV^33^. From the scattering probability data^33^, we estimate a maximum *p*-H_2_ to *o*-H_2_ ratio *R*_*J*=0/*J*=1_ of ca. 2 (as compared to ca. 4.96 and 16.1 along SrTiO_3_(001)[100] and SrTiO_3_(001)[110] of the TiO_2_- and SrO-terminated SrTiO_3_(001), respectively). (Note that the results of ref. ^33^ have since been confirmed experimentally^34^).

Considering the Debye temperature of SrTiO_3_ (ca. 413.3 K^35^), we may expect some thermal modulation/attenuation. With Ti having a smaller mass than Sr, we would expect more pronounced effect (modulation/attenuation) on TiO_2_-terminated SrTiO_3_(001), as compared to SrO-terminated SrTiO_3_(001). Consider for example a surface temperature of 100 K and incidence angle *Θ*_i_: [0°, 66°]. For TiO_2_-terminated SrTiO_3_(001), we estimated Debye-Waller factor values (cf., e.g., ref. ^12^ for more details) ranging from ca. 0.3 to 0.6, increasing with increasing incidence angle *Θ*_i_. In comparison, for SrO-terminated SrTiO_3_(001), we estimated^12^ Debye-Waller factor values ranging from ca. 0.6 to 0.8, increasing in value with increasing incidence angle *Θ*_i_. The modulation/attenuation would become more pronounced with increased temperature. However, regardless of the degree of modulation/attenuation, the maximum *p*-H_2_ to *o*-H_2_ ratio for each surface termination remained almost the same (viz., ca. 4.48 and 16.7 along the SrTiO_3_(001)[100] and SrTiO_3_(001)[110] of the TiO_2_- and SrO-terminated SrTiO_3_(001), respectively, at 100 K).

Finally, because of the wide spread of the scattering angle, we could expect small scattering probabilities at each particular scattering angle. Furthermore, the larger the (surface lateral) incident energy, the more number of (surface lateral) diffraction channels (would be) involved (excited). As a result, the normalized *p*-H_2_ to *o*-H_2_ ratio $\bar{{R}_{J=0/J=1}}$ shows small values (cf., Tables 1 and 2). Thus, so far, we can collect only a small amount of H_2_ through any given scattering event. (But with high purity!) For engineering applications, scattering at optimum surface angles should resolve this problem.

## Methods

### Model Hamiltonian

To study the dynamics of H_2_ scattering on STO(001) (Fig. 1), we performed quantum dynamical calculations (cf., e.g., refs. ^1–5,8,9,24,31,32^) by solving the corresponding time-independent Schrödinger equation for H_2_, in the vibrational ground state (*ν* = 0), under the influence of an orientationally anisotropic potential energy (hyper-) surface (PES), using the coupled-channel method^36–42^. The dynamical variables we considered include the H_2_ center-of-mass (CM) distance *Z* from the surface, the H_2_ bond-length *r*, the polar and azimuthal angular orientations of the H-H bond with respect to the surface, *θ* and *ϕ*, respectively, and the position of the H_2_ CM *X*, along STO(001)[100] and STO(001)[110] of the TiO_2_- and SrO-terminated STO(001), respectively. Considering that the energy scale of the H_2_ molecular vibration (*ℏω* = 516 meV) exceeds the energy range relevant to our current study (i.e., *E*_i_: [10, 80] meV), we can neglect the molecular vibrational excitations, and fix the H_2_ interatomic distance at *r* = 0.74 Å. The small variation of the potential energy with respect to *ϕ* allows us to further neglect the *ϕ*-dependence^24^. Thus, we can reduce the original 6-Dimensional (6-D) Hamiltonian (for the diatomic molecule-surface system) to the following simplified 3-D form :

1 $$H=-\frac{{\hslash }^{2}}{2m}\left(\frac{{{\rm{\partial }}}^{2}}{{\rm{\partial }}{Z}^{2}},+,\frac{{{\rm{\partial }}}^{2}}{{\rm{\partial }}{X}^{2}}\right)-\frac{{\hslash }^{2}}{2I}\left[\frac{1}{\sin \theta },\frac{{\rm{\partial }}}{{\rm{\partial }}\theta },(\sin \theta \frac{{\rm{\partial }}}{{\rm{\partial }}\theta }),+,\frac{1}{{\sin }^{2}\theta },\frac{{{\rm{\partial }}}^{2}}{{\rm{\partial }}{\phi }^{2}}\right]+V(Z,\theta ,X).$$

*m* and *I* correspond to the H_2_ total mass and moment of inertia, respectively.

### Potential Energy (Hyper-) Surface: PES

The 3-D PES *V*(*Z*, *θ*, *X*) in Eq. (1) comes from previously performed density functional theory (DFT)-based total energy calculations^43–45^ for H_2_ adsorption at the Ti and O surface sites on the TiO_2_-terminated STO(001), the Sr and O surface sites on the SrO-terminated STO(001), and H_2_ polar orientations *θ* = 0 and *π*/2^24^. We fitted the potential energy curves for each configuration using Morse potentials:

2 $${V}_{{\rm{A}}({\rm{B}}),\theta }={D}_{{\rm{A}}({\rm{B}}),\theta }\times \{\exp [-\,2{\alpha }_{{\rm{A}}({\rm{B}}),\theta }(Z-{Z}_{{\rm{A}}({\rm{B}}),\theta }^{{\rm{e}}{\rm{q}}})]-2\exp [{\alpha }_{{\rm{A}}({\rm{B}}),\theta }(Z-{Z}_{{\rm{A}}({\rm{B}}),\theta }^{{\rm{e}}{\rm{q}}})]\},$$

and connected the Morse potentials for *θ* = 0 and *π*/2 at each surface site and for different surface sites to get

3 $$V(Z,\theta ,X)=[{V}_{{\rm{A}},\theta =\pi /2}{\sin }^{2}\theta +{V}_{{\rm{A}},\theta =0}{\cos }^{2}\theta ]{\cos }^{2}gX+[{V}_{{\rm{B}},\theta =\pi /2}{\sin }^{2}\theta +{V}_{{\rm{B}},\theta =0}{\cos }^{2}\theta ]{\sin }^{2}gX.$$

*D*_A(B),*θ*_, *α*_A(B),*θ*_, ${Z}_{{\rm{A}}({\rm{B}}),\theta }^{{\rm{e}}{\rm{q}}}$ give the corresponding potential depth, potential width, and equilibrium (normal/perpendicular) distance of H_2_ from the surface cation sites (A:[Ti, Sr]) and oxygen site (B), at H-H bond angle *θ*, respectively. The reciprocal lattice constant *g*_*X*_(= *π*/*a*_*X*_), with corresponding direct lattice constants ${a}_{X}^{[100]}=3.91$ Å and ${a}_{X}^{[110]}=5.52$ Å along [100] and [110] of the TiO_2_- and SrO-terminated STO(001), respectively^46^. Table 3 shows the fitted parameters for each configuration. We used spherical harmonics and plane waves as basis sets for the rotational motion and the translational motion (perpendicular to the surface and along the surface direction *X*), respectively.

**Table 3:** Fitted values of the potential depth *D*, potential width *α*, and equilibrium distance from the surface *Z*^eq^, at each surface site, in Eq. (3).

Surface	adsorption	*θ*	*D*	*α*	*Z*^eq^
Termination	site	[rad]	[eV]	[Å^−1^]	[Å]
TiO_2_	Ti site-A	0	0.0390	1.40	3.18
		*π*/2	0.172	1.47	2.37
	O site-B	0	0.0665	1.30	2.97
		*π*/2	0.0486	1.24	3.14
	Sr site-A	0	0.0409	1.21	3.57
SrO		*π*/2	0.108	1.39	2.93
	O site-B	0	0.152	1.18	2.55
		*π*/2	0.0322	0.755	3.70

To extract the contribution of the electrostatic interaction between the H_2_ quadrupole moment and the surface local electric field, as discussed above, we used the charge density distribution obtained from previous DFT-based total energy calculations^24^. We calculated the induced dipole Δ*D*_*i*_ (*i* = *x*, *y*, *z*) and quadrupole moments Δ*Q*_*i*,*j*_ (*i*, *j* = *x*, *y*, *z*) from the charge density difference ($\Delta \rho (Z)={\rho }_{{{\rm{H}}}_{2}/STO}(Z)-{\rho }_{{{\rm{H}}}_{2}}-{\rho }_{{\rm{STO}}}$) as a function of *Z* for the Ti and O sites (TiO_2_-termination) and Sr and O sites (SrO-termination) with *θ* = 0 and *π*/2. We also calculated the H_2_ quadrupole moment *Q*_*i*,*j*_ from the charge density distribution of the isolated system. We used the pristine STO(001) to calculate the surface electric field and its gradient.

### Scattering probability

Consider a H_2_ impinging with an initial rotational state (*J*_i_, *m*_i_), incident energy *E*_i_, and angle of incidence with respect to the surface normal *Θ*_i_ (cf., e.g., Fig. 1(c,d), and Table 4). Using the coupled-channel method^36–42^, we calculated the probability ${P}_{{J}_{{\rm{i}}}\to {J}_{{\rm{f}}}}^{{m}_{{\rm{i}}}\to {m}_{{\rm{f}}}}({E}_{{\rm{i}}},{E}_{{\rm{f}}},{\varTheta }_{{\rm{i}}},{\varTheta }_{{\rm{f}}})$ of finding the H_2_ scattered with a final rotational state (*J*_f_, *m*_f_), final kinetic energy *E*_f_, scattering at an angle of *Θ*_f_ with respect to the surface normal. (We carefully checked the convergence for calculations with maximum quantum numbers ${J}_{\max }=10$ and $| {G}_{{X}_{\max }}| =30$). From the calculated scattering probability ${P}_{{J}_{{\rm{i}}}\to {J}_{{\rm{f}}}}^{{m}_{{\rm{i}}}\to {m}_{{\rm{f}}}}({E}_{{\rm{i}}},{E}_{{\rm{f}}},{\varTheta }_{{\rm{i}}},{\varTheta }_{{\rm{f}}})$ of H_2_ on STO(001), we evaluated the corresponding *p*-H_2_ to *o*-H_2_ ratio ${R}_{{p-{\rm{H}}}_{2}(J=0)/{o-{\rm{H}}}_{2}(J=1)}({E}_{{\rm{i}}},{\varTheta }_{{\rm{i}}},{\varTheta }_{{\rm{f}}})={R}_{J=0/J=1}({E}_{{\rm{i}}},{\varTheta }_{{\rm{i}}},{\varTheta }_{{\rm{f}}})$, given the incident energy *E*_i_, incident angle *Θ*_i_, and scattering angle *Θ*_f_, i.e.,

4 $${R}_{J=0/J=1}({E}_{{\rm{i}}},{\varTheta }_{{\rm{i}}},{\varTheta }_{{\rm{f}}})=\frac{\int d{E}_{{\rm{f}}}\,\,{P}_{{J}_{{\rm{i}}}=0\to {J}_{{\rm{f}}}=0}^{{m}_{{\rm{i}}}=0\to {m}_{{\rm{f}}}=0}({E}_{{\rm{i}}},{E}_{{\rm{f}}},{\varTheta }_{{\rm{i}}},{\varTheta }_{{\rm{f}}})}{\int d{E}_{{\rm{f}}}{\sum }_{m=-1}^{1}{P}_{{J}_{{\rm{i}}}=1\to {J}_{{\rm{f}}}=1}^{{m}_{{\rm{i}}}\to {m}_{{\rm{f}}}={m}_{{\rm{i}}}}({E}_{{\rm{i}}},{E}_{{\rm{f}}},{\varTheta }_{{\rm{i}}},{\varTheta }_{{\rm{f}}})}=\frac{{P}_{0}^{0}({E}_{{\rm{i}}},{\varTheta }_{{\rm{i}}},{\varTheta }_{{\rm{f}}})}{{\sum }_{m=-1}^{1}{P}_{1}^{m}({E}_{{\rm{i}}},{\varTheta }_{{\rm{i}}},{\varTheta }_{{\rm{f}}})}.$$

Note that

5 $$\int \ d{E}_{{\rm{f}}}\,\sum _{{J}_{{\rm{f}}},{m}_{{\rm{f}}},{\varTheta }_{{\rm{f}}}}{P}_{{J}_{{\rm{i}}}\to {J}_{{\rm{f}}}}^{{m}_{{\rm{i}}}\to {m}_{{\rm{f}}}}({E}_{{\rm{i}}},{E}_{{\rm{f}}},{\varTheta }_{{\rm{i}}},{\varTheta }_{{\rm{f}}})=1.$$

And since we are considering the case when *J*_i_ = *J*_f_ and *m*_i_ = *m*_f_, in Eq. (4) we have

6 $${P}_{{J}_{{\rm{i}}}\to {J}_{{\rm{f}}}}^{{m}_{{\rm{i}}}\to {m}_{{\rm{f}}}}({E}_{{\rm{i}}},{E}_{{\rm{f}}},{\varTheta }_{{\rm{i}}},{\varTheta }_{{\rm{f}}})={P}_{{J}_{{\rm{i}}}\to {J}_{{\rm{f}}={\rm{i}}}}^{{m}_{{\rm{i}}}\to {m}_{{\rm{f}}={\rm{i}}}}({E}_{{\rm{i}}},{E}_{{\rm{f}}},{\varTheta }_{{\rm{i}}},{\varTheta }_{{\rm{f}}})={P}_{J}^{m}({E}_{{\rm{i}}},{\varTheta }_{{\rm{i}}},{\varTheta }_{{\rm{f}}}).$$

We also calculated the normalized *p*-H_2_ to *o*-H_2_ ratio $\bar{{R}_{J=0/J=1}}({E}_{{\rm{i}}},{\varTheta }_{{\rm{i}}},{\varTheta }_{{\rm{f}}})$, i.e.,

7 $$\bar{{R}_{J=0/J=1}}({E}_{{\rm{i}}},{\varTheta }_{{\rm{i}}},{\varTheta }_{{\rm{f}}})={R}_{J=0/J=1}({E}_{{\rm{i}}},{\varTheta }_{{\rm{i}}},{\varTheta }_{{\rm{f}}})\times \frac{{P}_{0}^{0}({E}_{{\rm{i}}},{\varTheta }_{{\rm{i}}},{\varTheta }_{{\rm{f}}})+{\sum }_{m=-1}^{1}{P}_{1}^{m}({E}_{{\rm{i}}},{\varTheta }_{{\rm{i}}},{\varTheta }_{{\rm{f}}})}{{\sum }_{{\varTheta }_{{\rm{f}}}=-9{0}^{\circ }}^{9{0}^{\circ }}[{P}_{0}^{0}({E}_{{\rm{i}}},{\varTheta }_{{\rm{i}}},{\varTheta }_{{\rm{f}}})+{\sum }_{m=-1}^{1}{P}_{1}^{m}({E}_{{\rm{i}}},{\varTheta }_{{\rm{i}}},{\varTheta }_{{\rm{f}}})]}.$$

In Eq. (7), we multiplied the angle specific *p*-H_2_ to *o*-H_2_ ratio by a normalization factor, so as to evaluate the efficiency of rotational state separation with respect to the incident *n*-H_2_. Note that the *p*-H_2_ to *o*-H_2_ ratio of *n*-H_2_ corresponds to 0.333 (1/3) at room temperature (*T* = 300 K)

**Table 4:** Some relevant physical values corresponding to H_2_(*E*_i_, *J*_i_, *m*_i_, *Θ*_*i*_) impinging a TiO_2_- and SrO-terminated SrTiO_3_(001). Incident angles presented in this work doubly underlined.

	TiO_2_-termination	SrO-termination
	${a}_{X}^{[100]}=3.91$ Å	${a}_{X}^{[110]}=5.52$ Å
	*E*_i_ = 80 meV	*E*_i_ = 60 meV
	${E}_{{\rm{i}}}^{{\rm{total}}}(J=0)=80.0$ meV	*E*_total_(*J* = 0) = 60.0 meV
	${E}_{{\rm{i}}}^{{\rm{total}}}(J=1)=95.2$ meV	*E*_total_(*J* = 1) = 75.2 meV
${G}_{{{\rm{i}}}_{X}}$	incident angle *Θ*_i_[°]	incident angle *Θ*_i_ [°]
0	0.00	0.00
1	5.26	4.29
2	10.6	8.60
3	$\underline{\underline{{15.9}^{\dagger }}}$	13.0
4	21.5	17.4
5	27.3	22.0
6	33.3	26.7
7	39.9	31.6
8	47.1	36.7
9	55.5	42.3
10	66.3	48.4
11	—	55.3
12	—	63.8
13	—	$\underline{\underline{{76.5}^{\ddagger }}}$

$${}^{\dagger }{E}_{\parallel }={E}_{X}={E}_{{\rm{i}}}{\sin }^{2}{15.9}^{\circ } \sim 6.004$$ meV.

$${}^{\dagger }{E}_{\perp }={E}_{Z}={E}_{{\rm{i}}}{\cos }^{2}{15.9}^{\circ } \sim 74.00$$ meV.

$${}^{\ddagger }{E}_{\parallel }={E}_{X}={E}_{{\rm{i}}}{\sin }^{2}{76.5}^{\circ } \sim 56.73$$ meV.

$${}^{\ddagger }{E}_{\perp }={E}_{Z}={E}_{{\rm{i}}}{\cos }^{2}{76.5}^{\circ } \sim 3.270$$ meV.

• *E*_i_: incident energy.

• (*J*_i_, *m*_i_): initial rotational state.

• *Θ*_i_: incident angle with respect to the surface normal.

• ${E}_{{\rm{i}}}^{{\rm{total}}}={E}_{{\rm{i}}}+{E}_{{J}_{{\rm{i}}}}$: total initial kinetic energy.

• ${E}_{{J}_{{\rm{i}}}}={B}_{{{\rm{H}}}_{2}}{J}_{{\rm{i}}}({J}_{{\rm{i}}}+1)$: initial rotational energy.

• ${B}_{{{\rm{H}}}_{2}}=7.6$ meV: H_2_ rotational constant.

• ${E}_{\perp }={E}_{Z}={E}_{{\rm{i}}}{\cos }^{2}\varTheta$: surface normal translational energy.

• ${E}_{\parallel }={E}_{X}={E}_{{\rm{i}}}{\sin }^{2}\varTheta =\frac{{\hslash }^{2}}{2M}{[g{G}_{{{\rm{i}}}_{X}}]}^{2}$: surface lateral translational energy.

• *M*: H_2_ total mass.

• $g=\frac{\pi }{{a}_{X}}$: reciprocal lattice constant.

• *a*_*X*_: direct lattice constant.

• ${G}_{{{\rm{i}}}_{X}}$: surface lateral wave number (integer).
